# Biotechnological sovereignty is not a mere nationalist concept, it is a necessity for Colombia and Latin America

**DOI:** 10.1590/0102-311XEN202323

**Published:** 2024-09-20

**Authors:** Camilo Guzman, Salim Mattar, Nelson Alvis-Guzman, Fernando De la Hoz, Edgar Arias

**Affiliations:** 1 Instituto de Investigaciones Biológicas del Trópico, Universidad de Córdoba, Montería, Colombia.; 2 Alzak Foundation, Cartagena de Indias, Colombia.; 3 Universidad de Cartagena, Cartagena de Indias, Colombia.; 4 Departamento de Salud Pública, Universidad Nacional de Colombia, Bogotá, Colombia.; 5 Instituto Nacional de Salud, Bogotá, Colombia.

**Keywords:** Pharmaceutical Economics, Biomedical Technology, Drugs Essential, Social Medicine, Economía Farmacéutica, Tecnología Biomédica, Medicamentos Esenciales, Medicina Social, Farmacoeconomia, Tecnologia Biomédica, Medicamentos Essenciais, Medicina Social

## Abstract

During the pandemic, Latin American countries suffered the collapse of their health systems. This was caused by the high demand for care of patients infected with SARS-CoV-2, which was added to the care of patients with other diseases. The significant increase in demand for health services caused medical and laboratory supplies to decline rapidly. The COVID-19 pandemic exacerbated a health crisis in several developing countries, mainly caused by insufficient systematic policies for integrating scientific knowledge. The current Colombian government must formulate a Biotechnological or Biosecurity Sovereignty Law that guarantees scientific autonomy, ensuring that Colombia is self-sufficient in Science, Technology, and Innovation. Colombian government should also focus on establishing and developing pharmaceutical chemical production by acquiring active chemical ingredients from other countries. This strategy could reduce the production costs and final prices of medicines, as well as generate high-level employment and wealth for the country. In this way, the Colombian government could prevent shortage of essential medicines and excessive price increases by commercial intermediation. In conclusion, the manuscript focuses on the lack of biotechnological sovereignty in Colombia. We propose a model of a Latin American Science and Technology ecosystem to achieve biotechnological sovereignty via state funding of research, strengthening universities, and fostering participation among private companies and Ministries of Science, Education, Trade, and Health. Scientific autonomy based on innovative processes that strengthen biotechnological independence can contribute to the economy by generating gross added value, creating high-quality employment, and facilitating the appropriation and social dissemination of knowledge, and cost reduction.

## Resumen

Durante la pandemia, los países latinoamericanos vieron colapsar sus sistemas de salud. Esto se debió a la alta demanda de atención para las personas contagiadas con SARS-CoV-2 y a la atención de pacientes con otros tipos de enfermedades. El aumento significativo de la demanda de los servicios de salud llevó a la rápida disminución de los suministros médicos y de laboratorio. La pandemia de la COVID-19 intensificó la crisis sanitaria en los países en desarrollo, principalmente por una política sistemática e insuficiente de apropiación del conocimiento científico. Si el actual gobierno colombiano formulara una Ley de Soberanía Biotecnológica o de Bioseguridad por la cual se garantizaría la autonomía científica, esto conduciría a una autonomía de Colombia en Ciencia, Tecnología e Innovación. El Gobierno de Colombia también debería centrarse en fomentar y desarrollar la producción de productos químicos farmacéuticos mediante el abastecimiento de ingredientes químicos activos de otros países. Esta estrategia favorece la reducción de costos de producción y del precio final de los medicamentos, además de generar empleos de alto nivel y riqueza para el país. De esta manera, no habría escasez de medicamentos esenciales, ni alzas excesivas de precios en la intermediación comercial. Pero, la realidad es que hay una débil soberanía biotecnológica en Colombia. En este texto se propone un modelo de ecosistema basado en Ciencia y Tecnología para que Latinoamérica logre la soberanía biotecnológica mediante el financiamiento estatal a la investigación en universidades, con la participación de entidades privadas y Ministerios de Ciencia, Educación, Comercio y Salud. La autonomía científica se basa en procesos de innovación que implican el fortalecimiento de la autonomía biotecnológica.

## Resumo

Durante a pandemia, os países latino-americanos sofreram o colapso de seus sistemas de saúde. Isso foi causado pela alta demanda de atendimento aos infectados pelo SARS-CoV-2, que somou-se, em paralelo, ao atendimento de pacientes com outros tipos de doenças. O aumento significativo na demanda por serviços de saúde levou à rápida diminuição dos suprimentos médicos e laboratoriais. A pandemia de COVID-19 acelerou, nos países em desenvolvimento, uma crise sanitária causada principalmente por uma política sistemática e insuficiente de apropriação do conhecimento científico. O atual governo colombiano deve formular uma Lei de Soberania Biotecnológica ou de Biossegurança que garanta a autonomia científica, o que leva à autonomia que garante que a Colômbia seja autossuficiente em Ciência, Tecnologia e Inovação. O governo da Colômbia também deve se concentrar em estabelecer e desenvolver a produção de produtos químicos farmacêuticos, adquirindo ingredientes químicos ativos de outros países. Essa estratégia reduz os custos de produção e o preço final dos medicamentos, além de gerar empregos de alto nível e riqueza para o país. Desta forma, não haveria escassez de medicamentos essenciais, nem estaríamos sujeitos a aumentos excessivos de preços por intermediação comercial. Em conclusão, o manuscrito foca na fracassada soberania biotecnológica na Colômbia. Propomos um modelo de ecossistema latino-americano de Ciência e Tecnologia para alcançar a soberania biotecnológica por meio do financiamento estatal do fortalecimento da pesquisa das universidades, com a participação de empresas privadas e Ministérios da Ciência, Educação, Comércio e Saúde. A autonomia científica é baseada em processos de inovação que fortalecem a autonomia biotecnológica.

## Introduction

When the World Health Organization (WHO) declared the pandemic in February 2020, governments worldwide needed to implement immediate sanitary measures to prevent and control the spread of the virus among their population, as well as reduce/mitigate the impact on infection and mortality rates. Since many low- and middle-income countries lack the skills and capacities to rapidly develop their technologies for opportune and accurate diagnostics, they were forced to adopt restrictive measures on mobility and social distancing, which led to the collapse of their economies and an increase in social suffering ^1^. During the pandemic, Latin American countries suffered the collapse of their health systems. This was caused by the high demand for care for those infected with SARS-CoV-2, which was added to the care of patients with other diseases. The excess demand for health services caused medical and laboratory supplies to decline rapidly. Shortage was a reality, thus imported molecular diagnostic equipment, supplies, and kits soon became scarce. Due to a historical lack of strategic planning in Latin America for accessing scientific knowledge, there are few possibilities to establish biotechnological markets for diagnostic methods for infectious agents, medicines, vaccines, and medical devices, among others.

The importation of inputs for the manufacture of pharmaceutical products, vaccines, medical devices, and reagents for the diagnosis of tropical infectious diseases, as well as the system focused on the assembly of components, may be affected by the trade rules imposed on the country by international trade treaties and the local currency devaluation against the dollar. In the economic model based on knowledge development, nations, organizations, and individuals compete in the strategic line of production, application, appropriation, and commercialization of knowledge. Latin-American talent is well-valued and recognized; however, how will intellectual heritage develop when everything is imported? Scientific autonomy is justified because it promotes progress and benefits society. Access to medicines and health supplies is essential to achieve total health coverage. The increase in costs and the scarcity of medicines are some issues faced by developing countries. Ensuring equitable access to health supplies in health systems is a great challenge today ^2^ ^,^ ^3^.

This work is part of an extensive analysis of research and health public policy emphasizing biotechnological sovereignty.

## Inequality, availability and access to biotechnologies, epidemiological impact of COVID-19 and biotechnological dependency

Access to vaccines in developing countries has been slow, and in Africa, for example, low vaccination rates against SARS-CoV-2 persist. Inequality in the availability and access to technologies and biotechnologies caused some low-income countries to suffer high rates of excess mortality from COVID-19. A systematic review and meta-analysis on excess mortality from COVID-19, which included 20 studies from 79 countries, found an overall excess mortality of 104.84 (95%CI: 85.56-124.13) per 100,000 inhabitants. South America was 30% higher than the global rate; developing countries held twice the rates of developed countries (135.80 vs. 68.08). Lower-middle-income and upper-middle-income countries (133.45 vs. 149.88, respectively) showed twice the excess mortality of high-income countries ^4^. In Colombia, excess mortality attributable to COVID-19 was found to range from 15%-20% ^5^. Of 13 countries in South America, Colombia was the third country with the highest number of deaths (n = 142,713) and the fifth with the highest number of deaths, 2,770/million inhabitants ^6^ ^,^ ^7^. Could this high lethality be avoided with good availability and access to biotechnologies?

On the other hand, low- and middle-income countries were crucial in the COVID-19 pandemic with the genetic information on SARS-CoV-2 mutations, which they selflessly registered on databases, including the GISAID, NCBI GenBank, Pangolin, and others. In 2022, by using the COVID-19 variants genomic data, the pharmaceutical company Pfizer designed the bivalent vaccine and earned around USD 34 billion ^8^. However, the company did not provide financial support or doses of bivalent vaccines to South Africa, where Omicron was first identified ^8^.

Global inequality must decrease, and knowledge must be recognized with credits in scientific publications and monetary returns for the efforts of scientists in laboratories. The WHO encourages the sharing of pathogen genome data to protect global public health. Sharing data on the pathogen genome is essential to prevent, detect, and respond to epidemics and pandemics at the national and international levels and is of global benefit ^9^.

## Did the investment of gross domestic product in Science, Technology, and Innovation affect mortality from COVID-19?

**Figure 1:**
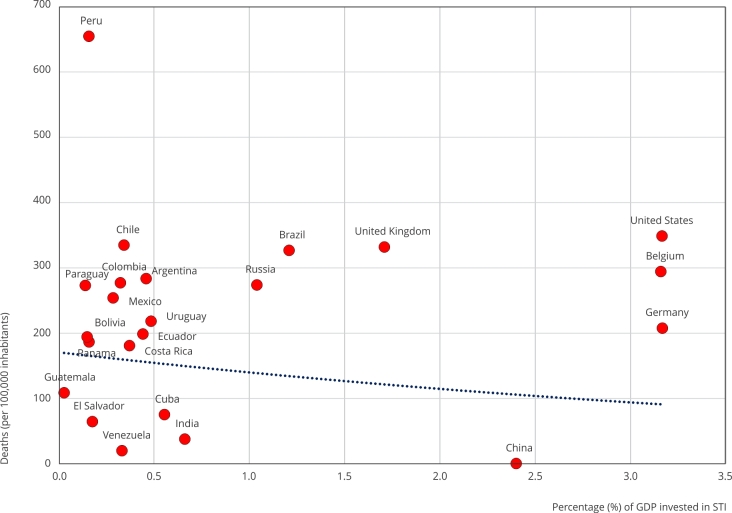
Comparison of mortalities rates of COVID-19 and percentage of gross domestic product (GDP) invested in Science, Technology, and Innovation (STI).

Source: GDP data obtained from the World Bank Open Data ^24^; mortality data per 100,000 inhabitants obtained from the Worldometer ^7^.

Gross domestic product (GDP) spending on Science, Technology, and Innovation (STI) does not follow a pattern proportional to COVID-19 mortality and GDP. Figure 1 shows an analysis of mortality and the percentage of GDP investment in STI for some Latin American and industrialized countries. In general, countries with higher investments in STI showed lower lethality per 100,000 inhabitants; however, this trend did not apply to all countries, as Peru showed the highest lethality in the world during the pandemic. Moreover, despite presenting a high investment in GDP, the United States, the United Kingdom, Belgium, and Russia held significant mortality rates. However, there is possibility of bias since some governments control public health statistics. Countries with little GDP investment in STI are unequal to others with high GDP investment. These inequalities were evident during the COVID-19 pandemic (Figure 1). In this sense, research and innovation constitute one of the most critical investments for competitiveness since it allows the generation of new knowledge to solve public health problems.

In contrast, Germany, India, and China did not have such high rates. On the Latin American side, GDP did not influence the high mortality rates except for Cuba. What happened in Brazil deserves a particular analysis, probably influenced by the political decisions of its rulers. Has the attitude of some led to neglect of public health? Let us remember that some erroneously recommended the use of ivermectin and underestimated care to avoid infection by the virus. Chile, despite the rapid start of vaccination of the population, suffered an inexplicable and significant mortality. The other Latin American countries showed similar behaviors; however, the first six countries of South America were above the rest of the Latin American countries. El Salvador showed an excellent epidemiological performance, and despite not manufacturing vaccines, it had the lowest mortality among the Latin American countries (Figure 1).

## Biotechnological development in the BRICS

The BRICS group, composed of Brazil, Russia, India, China, and South Africa, has played an essential role in the global vaccine market against COVID-19. These countries continue to increase the production capacity of vaccines to meet the high global demand of the modern world. The BRICS nations have successfully competed with pharmaceutical multinationals and have manufactured traditional vaccines initially included in the WHO’s Expanded Program on Immunization ^10^. In addition, by competing, they have also helped reduce prices in the United Nations and national vaccine markets. India supplies more than 50% of vaccines to the global pharmaceutical sector, meets 40% of the generic drug demand in the United States, and provides 25% of all medicines in the United Kingdom. India’s pharmaceutical industry holds a network of 3,000 companies and is vital in the global pharmaceutical sector.

During the SARS-CoV-2 pandemic, Russia developed a vaccine with recombinant RNA, the Sputnik, which was supplied and used to partly control mortality from COVID-19. For its part, China designed and manufactured several vaccines, including the chemically inactivated SARS-CoV-2 CoronaVac. China sold billions of vaccines at the start of the pandemic and contributed significantly to containing the spread of the virus, with Latin America and Asia purchasing millions of doses in 2021. China is 100% autonomous in producing its medicines ^11^. During the COVID-19 pandemic, pharmaceutical multinationals sold doses to Colombia and Latin America ^12^ on average for USD 12, inflated prices during such a health crisis.

## Biotechnological development in Cuba and Latin America

Regarding biotechnology, Cuba is the leader in the region, achieving a high COVID-19 vaccination rate in record time. This is the result of decades of scientific investment, research, discovery and innovation, professional training, and increased production capacity. Cuba shows decades of experience designing recombinant vaccines, including a national pediatric immunization program, with 98% of children under five years of age immunized against 13 diseases. Despite the embargo, Cuba currently produces 58% of its medicines, medical devices, and diagnostic reagents ^13^. During the pandemic, Cuba developed and manufactured three vaccines authorized for emergency use against SARS-Cov-2, including Soberana-02, Soberana-Plus, and Abdala. With its excellent preparation and biotechnological development, Cuba managed to control the pandemic in its territory and even export vaccines to other countries. However, it is unknown if Cuba continues to export vaccines to other countries such as Iran.

In contrast, other Latin American countries have struggled to note the importance of biotechnological autonomy and sovereignty, leading to shortages of biologicals and exaggerated prices. For instance, Brazil, in the 1980s, produced 55% of its pharmaceutical products; currently, it imports 95% of its medicines and 100% of its COVID-19 vaccines ^14^. In this context, the WHO supported an initiative to expand equitable access to COVID-19 health products. This initiative provided a global platform for developers to share knowledge, intellectual property, and data ^15^.

## Preferential treatment to obtain vaccines and technological restrictions wrongly promoted by Latin American countries

During the COVID-19 pandemic, rich countries had priority access to vaccines. this “VIP” treatment was caused by the monopoly held by large pharmaceutical companies that distributed the vaccines. Similarly, in 2009, during the influenza A/H1N1 pandemic, wealthy countries also monopolized vaccines, leading to shortages in biologics to control A/H1N1 ^16^. In 2009, the late availability of vaccines did not significantly impact developing countries since the severity of the disease was not as pronounced and the most susceptible group, composed of older adults, had some level of protection. In contrast, the delayed provision of a protective immunological memory against SARS-CoV-2 led to an unjustified loss of lives.

In Colombia, scientific autonomy and biotechnological self-sufficiency have been restricted for over 45 years to favor commercial interests that have not benefited the nation. The country’s health community still remembers the wrong decision to close the Colombian National Institute of Health (INS, acronym in Spanish) biological production plant at the end of the 1990s. This erroneous form of government suffocated scientific autonomy since it did not consult with the country’s researchers to agree on this decision, leading to deaths and cost overruns due to the need to import biologicals, mainly from Brazil in subsequent times ^17^. Adopting a free trade model does not justify the loss of society’s autonomy or the dependence of Colombian scientists on imported inputs. On the contrary, the Colombian State must promote sustainable research and the development of scientific and biotechnological sovereignty.

The pandemic highlighted the lack of investment in investment in research, development, and innovation activities in the lower-middle-income countries of Latin America (Figure 1), revealing an imbalance in the levels of competitiveness. Colombia has paid dearly in the pandemic for depending on other countries ^3^. Latin American countries must develop strategies and initiatives focused on research, development, and innovation activities at various maturity levels. This approach could allow increasing the number of patents and biotechnological developments to better prepare the region to handle universal public health crises.

## Challenges and prospects for overcoming the biotech lag and building a biotech ecosystem

Significant social, economic, and environmental achievements can be obtained if Latin America decisively embraces the challenge of establishing integrated health and STI policies that lead to biotechnological sovereignty. Currently, a slowdown in the Latin American economy can be noted, which poses considerable social challenges. During the pandemic, monetary poverty in countries like Colombia increased from 35.7% in 2019 to 39.3% in 2021, as well as an increase to 44.6% in rural areas ^18^.

In the international context, low economic growth and high inflation, coupled with untimely and volatile changes in the world market, are major concerns. This scenario can hinder access to medicines that are vital for maintaining health and quality of life. Colombia’s current pharmaceutical policy ^17^ focuses on the surveillance and development of commercial activities but lacks a pharmaceutical industrial policy, presenting no industrial autonomy to produce its medicines. For Colombia and Latin America, an autonomous pharmaceutical industry is necessary and can be achieved via investments in STI. Such an industry could enable Latin America to approach a competitive environment for developing and manufacturing its medicines. It is reasonable to think of a large international biotechnological consortium among Latin American countries that produce essential drugs, diagnostic medical supplies, and vaccines. In this sense, the Community of Latin American and Caribbean States (CELAC) and regional commercial markets could serve as a platform to launch and achieve biotechnological sovereignty in Latin America.

**Figure 2:**
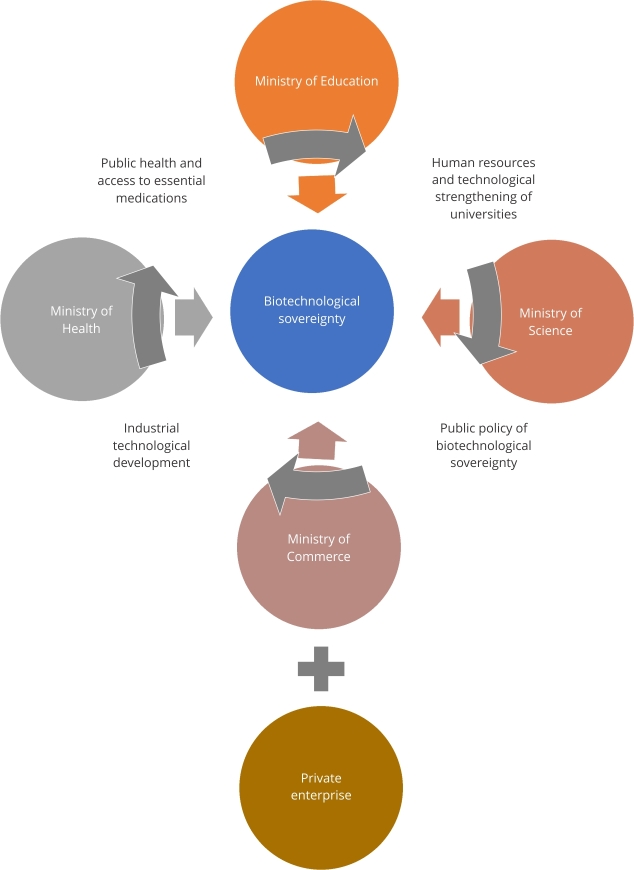
Proposed model of the Latin American ecosystem to achieve biotechnological sovereignty.

Biotechnological sovereignty is not merely a nationalist concept; it is an ecosystem that would allow the availability of medicines at a lower cost and could be achieved via coordinated policies from the Ministries of Health, Commerce, Science, and Education (Figure 2). With state support and a solid pharmaceutical policy, Colombia could manufacture widely used medicines to treat prevalent diseases (Figure 2), including antihypertensives, analgesics, hypoglycemic agents, antibiotics, antiretrovirals, anticancer drugs, and insulin. Biotech sovereignty is not a dream for third-world countries. In 2022, the French government announced an ambitious plan to produce at least 20 biotech products, including vaccines, with the entire project funded by the State Bank of France ^19^.

Colombia must develop and produce its medical supplies and technologies. The research and design of this ecosystem can be achieved with the support and encouragement of the Ministries of Health, Education, Commerce, and Science and Technology. Such a policy could encourage research into new therapeutic strategies for the most prevalent diseases in Colombia (Figure 2).

## The role of Latin American universities in implementing a biotechnological ecosystem

Recent world university rankings highlight improvements in the knowledge of Latin American universities. Brazil, Mexico, Chile, Argentina, and Colombia have generated impactful research publications that could be focused on and articulated in the development of pharmaceutical products. In other words, to achieve biotechnological and pharmaceutical sovereignty, Colombia already shows an ecosystem, as 97% of its research is conducted in public and private universities. The 32 public universities in Colombia account for 62% of the country’s STI activities. By associating with the INS, they could become essential actors for biotechnological and pharmaceutical sovereignty. In other words, the resources that public universities receive from the state could ensure a rapid return to society. Unfortunately, the average GDP allocation for STI in Colombia has not exceeded 0.25% over the last decade, which is far below the average levels of the Organization for Economic Cooperation and Development (OECD). With such a low GDP, Colombia will not be able to overcome these current challenges and will continue under external biotechnological submission. Moreover, with these poor investments, the country cannot approach industrial globalization or emerging technologies ^20^.

However, despite challenges in obtaining research resources, the Colombian academic community in biotechnology is competent, as demonstrated during the pandemic. Thanks to research groups from various universities in the country, it was possible to analyze up to 100,000 COVID-19 samples daily. In that process, Colombian researchers developed numerous innovations thanks to the funding and support to overcome the usual administrative and financial barriers. If the state were to establish a biotechnological and pharmaceutical sovereignty policy, it could rely on universities to develop projects addressing the needs for medicines, vaccines, and diagnostic supplies and devices. Establishing such a policy would aid develop a national pharmaceutical industry based on the valuable academic capital of Colombia (Figure 2). For a national biotechnological ecosystem, Colombia must be open and internationally oriented ^21^. In this sense, private companies could contribute with their capital to support the policy of biotechnological sovereignty.

## Private and public investment must coexist for the biotechnological development of Latin America

An example of biotechnological sovereignty in Colombia is Vecol, a company established over 65 years ago with the state as the majority shareholder. Under international quality standards, Vecol produces 12 vaccines for immunoprevention in veterinary medicine, as well as antibiotics and other agricultural products. We highlight that the main reason for the continued success and strengthening of the company’s biological production, beyond the quality of its processes, is due to the participation of private economic unions in its financing and operation, as well as the fact that most of its inputs are used by the production industry.

As the private company does, biotechnological sovereignty in Colombia can be an immediate reality via the development of pharmaceutical chemical production by acquiring active chemical ingredients from other countries. This strategy would reduce production costs and the final price of medicines while generating high-level employment and wealth for the country. In this way, shortages of essential medicines and excessive price increases could be avoided by reducing dependency on commercial intermediation and the private pharmaceutical industry. Undoubtedly, pharmaceutical and biotechnological sovereignty would facilitate access for the entire population and support the consolidation of “Health as a Fundamental Right”. In Colombia, as in most developing countries, there are concerns about the need for strategies to facilitate access to health services for neglected diseases and their populations. For these rare and expensive diseases, it is urgent to formulate policies that focus on the need to develop research to meet national demands.

Colombia requires a pharmaceutical industrial policy for the national, independent, sustainable production and development of medicines and other technologies. As established by Goal 17 the WHO’s Sustainable Development Goals ^21^, alliances are needed for productive development and investment in the public sector ^22^ ^,^ ^23^.

## Biodiversity is an advantage from nature to Latin America

Latin American countries benefit from rich biodiversity and agriculture resources but they are still underutilized. These natural resources represent a reservoir that requires increased investment to explore new promising species with potential compounds for pharmaceutical applications. Due to its remarkable biodiversity, Latin America shows opportunities for genomic surveillance of zoonotic and arthropod-transmitted viruses via animal reservoirs and vectors, which are crucial to understanding the context of epidemics ^8^. Universities and their research groups in Latin America can develop new synthetic molecules, showing promising results from in vitro and in vivo studies for treating some neglected tropical diseases. New biotechnological platforms would reduce costs and increase benefits, transforming the current scenario towards sanitary autonomy and allowing all territories greater disease control. It is important to note that SARS-CoV-2 could reemerge with new variants, requiring a updated vaccines. Then, if the current scenario persists, Colombia and other Latin American countries would have to line up again and pay for expensive doses of vaccines, as happened in 2021.

## Formulation of biotechnological sovereignty policies and conclusions

Technological dependence leads to vulnerability to epidemiological challenges and the influence of global pharmaceutical capital. To address this issue, the Colombian government should formulate a Biotechnological or Biosecurity Sovereignty Law that guarantees scientific autonomy, ensuring that Colombia is self-sufficient in STI. In conclusion, the state must promote and guarantee funding for research to develop vaccines, ophidian and scorpion antisera, diagnostic devices, and medicines, especially for orphan diseases and those with a lack of commercial interest. Moreover, developing technologies to produce medicines can aid mitigate the high prices in the world market. Scientific autonomy based on innovation processes that strengthen biotechnological autonomy contributes to the economy via increased gross added value, high-quality employment, and appropriation and dissemination of knowledge to society. Additionally, it reduces health costs and maintains high quality standards, thus benefiting patients by providing accessible technologies for diagnosing and treating prevalent diseases in the territory.

## References

[1] Mejia D, Diaz M, Charry A, Enciso K, Ramírez O, Burkart S (2021). "Stay at home": the effects of the COVID-19 lockdown on household
food waste in Colombia.. Front Psychol.

[2] Organización Mundial de la Salud Fondo estratégico. Acceso a medicamentos e insumos de salud de calidad
en las Américas. Informe Anual 2016..

[3] Guzman C, Mattar S, Alvis-Guzmán N, De la Hoz F (2023). The high price that Colombia has paid for its lack of
biotechnological sovereignty. Lancet Reg Health Am.

[4] Shang W, Wang Y, Yuan J, Guo Z, Liu J, Liu M (2022). Global excess mortality during COVID-19 pandemic a systematic
review and meta-analysis. Vaccines (Basel).

[5] Msemburi W, Karlinsky A, Knutson V, Aleshin-Guendel S, Chatterji S, Wakefield J (2023). The WHO estimates of excess mortality associated with the
COVID-19 pandemic. Nature.

[6] Cifuentes-Faura J (2021). COVID-19 mortality rate and its incidence in Latin America
dependence on demographic and economic variables. Int J Environ Res Public Health.

[7] Worldometer Reported cases and deaths by country or territory..

[8] Hill V, Githinji G, Vogels CBF, Bento AI, Chaguza C, Carrington CVF (2023). Toward a global virus genomic surveillance
network. Cell Host Microbe.

[9] World Health Organization WHO guiding principles for pathogen genome data sharing..

[10] Kaddar M, Milstien J, Schmitt S (2014). Impact of BRICS' investment in vaccine development on the global
vaccine market. Bull World Health Organ.

[11] Xue Y, Shang L (2022). Towards better governance on biosafety and biosecurity China's
advances and perspectives in medical biotechnology
legislation. Front Bioeng Biotechnol.

[12] Ávila Jiménez CC (2002). Esto es lo que pagó Colombia por las vacunas contra el
COVID-19.. El Tiempo.

[13] González CAG, Fonte DM, Rodríguez LL, González AG, Gomes JO (2022). Government strategies in confronting COVID-19 in the republic of
Cuba. Work.

[14] Mercadante E, Paranhos J (2022). Pharmaceutical patent term extension and patent prosecution in
Brazil (1997-2018). Cad Saúde Pública.

[15] World Health Organization WHO and MPP announce the first transparent, global, non-exclusive
licence for a COVID-19 technology..

[16] Hunter DJ, Abdool Karim SS, Baden LR, Farrar JJ, Hamel MB, Longo DL (2022). Addressing vaccine inequity - COVID-19 vaccines as a global
public good. N Engl J Med.

[17] Gomez-Marin JE (2020). Autonomía farmacéutica y biotecnológica frente a emergencias
sanitarias. Infectio.

[18] Departamento Nacional de Estadística Pobreza monetaria..

[19] Sharp PA, Patris J (2022). Para una innovación biotecnológica exitosa.. El Economista.

[20] Organisation for Economic Co-operation and Development Science, Technology, and Innovation Scoreboard..

[21] United Nations Development Programme Sustainable Development Goals..

[22] United Nations Secretary General's High-level Panel on Access to
Medicines Promoting innovation and access to health technologies..

[23] Pan American Health Organization Special Program, Innovation and Regional Production Platform
(RP)..

[24] World Bank Group World Bank Open Data..

